# Cultural adaptation of the guidelines for offering mental health first aid to a person after a potentially traumatic event: a delphi expert consensus study in Brazil

**DOI:** 10.1186/s12888-022-04269-4

**Published:** 2022-10-27

**Authors:** Kathlen Nataly Mendes, Carlos Henrique Mesquita Peres, Amanda Vidotto Cerqueira, Thais Alves Assumpção, Alexandre Andrade Loch, Nicola J Reavley

**Affiliations:** 1 Laboratorio de Neurociencias (LIM 27), Instituto de Psiquiatria, Hospital das Clinicas HCFMUSP, Faculdade de Medicina, Universidade de Sao Paulo, Sao Paulo, Brazil; 2 Instituto Nacional de Biomarcadores em Neuropsiquiatria (INBION), Conselho Nacional de Desenvolvimento Cientifico e Tecnológico, Sao Paulo, Brazil; 3 Centre for Mental Health, Melbourne School of Population and Global Health, University of Melbourne, 3010 Melbourne, VIC Australia

## Abstract

**Background:**

Traumatic events increase the risk of mental disorders. In a country with relatively under-developed mental health support systems, services to assist people who have experienced potentially traumatic events may be unavailable. In such situations, people in the community become key sources of support. However, they do not always have the knowledge and skills to offer effective help. This study reports on the cultural adaptation for Brazil of the English-language mental health first aid guidelines for helping someone who has experienced a potentially traumatic event.

**Methods:**

A Delphi expert consensus study with two expert panels, one comprising health professionals with experience in the treatment of trauma (n = 33) and the other comprising people with lived experience, (n = 29) was conducted. A questionnaire containing 131 statements from the English language guidelines was translated into Brazilian Portuguese. Participants were asked to rate the importance of actions to be taken to help a person who has experienced a potentially traumatic event and to suggest new items where appropriate.

**Results:**

Data were collected over two survey rounds. A total of 149 items were included in the final guidelines (110 items from the English-language guidelines and 39 new items created from expert panel comments, in the second round). Immediate action items were endorsed by both panels, while items related to encouraging victims were rejected by the professional panel. The suggested statements mostly related to providing psychological support and attending to the person’s subjective experience rather than providing material or structural support.

**Conclusion:**

While there were many similarities with the English-language guidelines for high-income countries, the guidelines also incorporate actions of importance for Brazil, including the emphasis on the first aider’s management of the person’s subjective experiences. These guidelines may inform Mental Health First Aid training for Brazil and may also be used as standalone resources.

**Supplementary information:**

The online version contains supplementary material available at 10.1186/s12888-022-04269-4.

## Introduction

Any event in which a person experiences or witnesses actual or threatened death, serious injury, or sexual violence is potentially traumatic [1]. Most people who experience such an event will be emotionally affected, with people responding in different ways. Use of the term ‘potentially traumatic event’ emphasizes the varied impacts; an event may have relatively little impact on one person, while in others, such events may cause anxiety, depression, acute stress disorder (ASD), posttraumatic stress disorder (PTSD), or other diagnosable mental illnesses [2]. Potentially traumatic events include large-scale situations such as disasters — natural or human-caused — or individual incidents such as being robbed or kidnapped [3]. In the context of the increasing urbanization seen in many countries and the impact of human-induced climate change, traumatic events are becoming increasingly common and greater attention is being paid to addressing the mental health consequences of experiencing a traumatic event [4–6]. This is especially true in developing countries, in which disasters often hit an unprepared society, and in which chaotic urbanization frequently leads to higher levels of violence, both of which impact mental health [7]. Furthermore, the recent COVID-19 pandemic and related social impacts may also be a source of mass trauma to the general population [8].

In recent years in Brazil, growing concern about the impact of natural disasters such as floods, droughts, and landslides as well as other potentially traumatic events has led to an increased focus on the health impacts, including the risk of mental disorders [9]. Recent events in the country have fueled the concern regarding this issue. These include the Kiss Nightclub fire (2013), in which the high death toll (over 230 people) was linked to poor compliance with safety regulations; the “Ninho do Urubu”— a soccer’s team lodging — fire (2019), the Mariana and Brumadinho dam disasters in 2013 and 2019 respectively. Research conducted in the aftermath of these disasters has highlighted the need for effective early intervention to help reduce the risk of developing long term mental health problems in those exposed to the effects of disasters [10–12]. Further adding to the risk of mental disorders such as PTSD, rates of interpersonal violence are high in Brazil. With the world’s ninth highest murder rate (31.1 people killed per 100,000 inhabitants), Brazil is considered one of the most violent countries in the world [13]. Experiencing violence is a risk factor for common mental disorders including depression, anxiety, and substance use, including in young people [14].

When exposure to potentially traumatic events occurs, timely and appropriate intervention may reduce the risk of developing mental health problems or reduce the severity of illness that does develop [15]. However, many of those at risk do not receive appropriate support or treatment, even if services are available. There is growing evidence that family and friends can play important roles in recognizing when a person is showing the symptoms of mental health problems, providing support and, if needed, encouraging the﻿ person to seek professional help. However, in general, lay people do not have the appropriate knowledge, skills or confidence to provide help [16, 17].

In an effort to address this problem, training programs and guidelines have been produced, including the WHO Guidelines for Psychological First Aid [18], which was developed in 2011 and provides general guidance to a broad range of countries. Another intervention that can be used to upskill members of the public responding to mental health crises, is the Mental Health First Aid (MHFA) training program, which was founded in Australia in 2001 [19]. It aims to teach course participants how to recognize when someone is developing a mental health problem or in a crisis and to assist them by providing ‘mental health first aid’, which has been defined as: “The help offered to a person developing a mental health problem, experiencing a worsening of an existing mental health problem or in a mental health crisis.” The first aid is given until appropriate professional help is received or until the crisis resolves. Since its inception, the course has spread to over 30 other countries and over 4 million people have been trained globally [20]. A recent meta-analysis that included 18 randomized controlled trials (RCTs) showed that MHFA training reduced stigmatizing attitudes and improved mental health first aid knowledge, recognition of mental disorders, beliefs about effective treatments and confidence and intentions to help a person with a mental health problem. There were also improvements in the help provided to a person with a mental health problem [16].

MHFA training is based on guidelines developed through the Delphi expert consensus method, involving panels of health professionals and people with lived experience of mental health problems. However, these guidelines development studies have involved mental health professionals and people with lived experience from high-income countries [21, 22] and their appropriateness for use in a country such as Brazil, with different culture and health systems, is unknown [23]. This includes the guidelines on how to assist a person who has experienced a potentially traumatic event as these were originally developed for use in high-income country contexts [17]. Therefore, the aim of this study was to use the Delphi expert consensus method to culturally adapt mental health first aid guidelines for assisting a person who has experienced a potentially traumatic event that are culturally appropriate for Brazil.

## Methods

The study involved four stages: (1) questionnaire development, (2) panel recruitment and formation, (3) data collection and analysis, (4) guidelines development.

### The Delphi method

The survey was conducted using the Delphi method, a systematic way of assessing the degree of consensus between groups of experts. In this study, use of the method relied on assessing the agreement between two panels of experts who were asked to rate the importance of specific actions for inclusion in guidelines for helping a person who had experienced a potentially traumatic event [24].

### Development of the questionnaire

The first step involved translation and adaption of the statements from the questionnaire used to develop the 2009 English-language mental health first aid guidelines on how to help someone who has experienced a potentially traumatic event to Brazilian Portuguese. This was done by a senior psychiatrist (AAL) and three medical students (CHMP, TAA, ACV). The translated version was then checked by AAL and sent to 10 individuals with a high language proficiency level to check for inconsistencies and readability. The 131 statements were then added to an online survey website (SurveyMonkey) and grouped into the following categories: actions to be taken immediately; recommendations for communication with the traumatized person; talking about the trauma; immediate assistance for large-scale traumatic events; coping strategies: chatting; coping strategies: actions; when to seek professional help; helping a traumatized child; attending to children in large scale traumatic events; communication with a traumatized child; if the first aider lives with a traumatized child; dealing with avoidance behaviors and tantrums; legal issues related to child abuse; getting professional help for a traumatized child.

### Recruitment of participants

Two expert panels were recruited to complete the questionnaire. One consisted of mental health professionals with expertise in trauma, and the other was composed of lay people with lived experience of trauma or with experience supporting a person who had experienced trauma. Health professionals were recruited through hospitals, community mental healthcare centers and universities, including the Institute of Psychiatry of the University of São Paulo. This also included academic mental health professionals’ groups specializing in the treatment of PTSD. They were approached personally, by e-mail or by telephone. For the lived experience panel, members of community support groups were invited, as well as patients with lived experience, including survivors of the recent catastrophes in Brazil named above. They were recruited from support groups in social media platforms (Facebook, websites, etc.) and from specialty mental health services dedicated to people who have experienced disasters.

Brief information about the study was presented to all participants along with a survey hyperlink, containing further information about the survey. Before starting the questionnaire, participants needed to consent to participation by checking a box. Participants could complete the survey in multiple sittings and in any location they desired. The participants had to be aged 18 years or older. The study was approved by the University of Melbourne and University of Sao Paulo ethics committees.

### Data collection and analysis

Participants were asked to rate each statement according to how important they believed it was for inclusion in the guidelines for providing mental health first aid to a person after a potentially traumatic event. We used a 5-point scale with the following response options (‘essential’, ‘important’, ‘not important’ ‘should not be included’ and ‘depends/don’t know’). At the end of each section, an open-ended text box was included, and participants were asked to suggest additional statements if they wished.

After the first round, statements were immediately included in the guidelines if they were endorsed by ≥ 80% of members in both panels as either essential or important. Statements were re-rated in the following round if they were rated as essential or important by 70–79% of either panel. Statements were immediately excluded from the guidelines if they were rated as essential or important by less than 70% of any panel. These percentage levels are in line with previous Delphi studies to culturally adapt mental health first aid guidelines [24, 25].

Items allocated to the ‘re-rate’ group were included in the second round, as well as new items based on any of the open-ended comments made by the participants that represented new ideas. After the second round, statements that received at least 80% ‘essential’ and ‘important’ ratings from both panels were endorsed, while the remaining statements were rejected. All endorsed items constituted than the final guidelines for the Brazilian guidelines for helping a person at risk of a traumatic event.

## Results

### Participants

For the first stage of the study, 62 participants were recruited, 33 mental health professionals and 29 people with lived experience of traumatic events. (See Table 1). Of these, 39 also responded during the second stage, that is, 27 professionals and 12 people with lived experience. The retention rate was 62.9% from the first round to the second. First round participants were aged between 18 and 64 years (mean = 35.2 years); 46 (74.2%) were women and 16 (25.8%) were men and all were born in Brazil where they currently reside. Among the professionals, 18 (54.6%) were psychologists, 6 (18.2%) nurses, 4 (12.2%) psychiatrists, 3 (9.0%) researchers and 2 (6.0%) volunteers (including health professional students).

**Table 1:** Characteristics of participants

Characteristics of participants	First round (n = 62)	Second round (n = 39)
**Sex**		
Female, n (%)	46 (74.2%)	30 (76.9%)
Male, n (%)	16 (25.8%)	9 (23.8%)
Age, Mean (SD) Range	35.2 (11.4) 18 to 64 years	33.5 (9.9) 18 to 59 years
**Profession (professional panel)**	**(n = 33)**	**(n = 21)**
Psychologists	18 (54.6%)	10 (47.7%)
Nurses	6 (18.2%)	2 (9.5%)
Psychiatrists	4 (12.2%)	4 (19.0%)
Researchers	3 (9.0%)	3 (14.3%)
Volunteers	2 (6.1%)	2 (9.5%)
**Source of experience (lay panel)**	**(n = 29)**	**(n = 12)**
Personal experience	22 (75.9%)	3 (25.0%)
Familial experience / caregiver	7 (24.1%)	9 (75.0%)

### Ratings of the statements

**Fig. 1:**
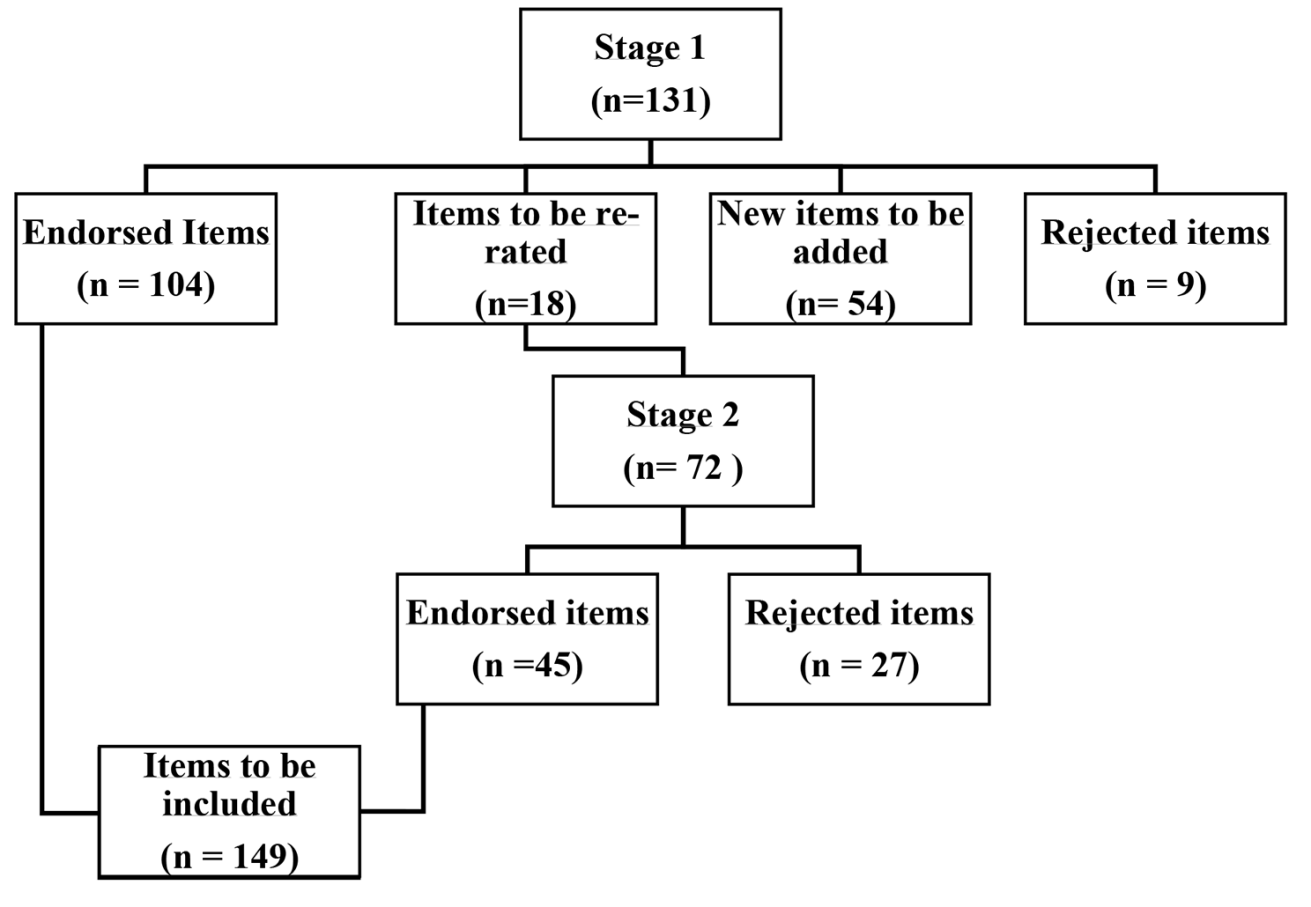
Overview of accepted and rejected statements

In the first round there were 131 statements, 104 of which were immediately endorsed from the English guidelines, 18 re-rated and 9 rejected (see Fig. 1). Fifty-four new statements were added after Round 1. In round 2, 6 of the re-rated items from the English language guidelines were endorsed and 39 of the 54 newly added items were endorsed. The changes relate to 11 out of the 14 sections of the round 1 questionnaire.

### Overview of sections and comparison between panels

Of the 131 initial statements, 82.4% (n = 108) of items had ratings with differences of 10% or less. Of these 87.0% (n = 94) were endorsed, 10.2% (n = 11) were re-rated, and 2.8% (n = 3) were excluded in the first round by both panels. The sections with the least differences were the following: Actions to be taken immediately; When to seek professional help; Children at large-scale traumatic events.

Of those items that had endorsement rating differences of more than 10% in round 1, those that received higher ratings from people with lived experience, tended to view the autonomy of the person being helped somewhat differently. Some items, such as e.g. “The first aider should communicate with the person as an equal, rather than as a superior expert. “and “The first aider should ask the person how they would like to be helped”, suggested a view that the person should have autonomy, while some other items received higher ratings from health professionals, e.g., “The first aider should avoid saying things which minimise the person’s feelings, such as “don’t cry” or “calm down” and “The first aider should not tell the person how they should be feeling.” These differences may relate to the first aider’s closeness to the person and their role in supporting them in their life.

Another area of divergence between the panels, involved the guidelines with children. Health professionals endorsed more highly the items involving the search for professional help while people with lived experience were more likely to endorse confronting a perpetrator.

### Additional items suggested by Brazilian panel

In general, the suggestions of the Brazilian participants focused on more psychological and emotional aspects of supporting a person experiencing a potentially traumatic event, with the concern about the first aider and his/her limits, stressing the importance of the first aider also receiving assistance when necessary. New items also included those relating to the need for careful, calm, attentive, and welcoming approaches to both adults and children. For children, new items focused on the need to establish a safe bond, provide security, welcome, not to doubt what the child says, and not lie to the child. The use of play was also mentioned as a useful strategy for engaging with children.

## Discussion

To the best of our knowledge, this is the first cultural adaptation of the guidelines for helping a person at risk of a potentially traumatic event for any country in Latin America. This is the first Brazilian study to culturally adapt the English-language MHFA guidelines based on Delphi expert consensus for assisting people in crisis. There have been several studies in English-speaking countries, which have higher economic status and relatively well-resourced mental healthcare systems, making it easier to conduct such studies [16, 26, 27]. However, such research is less common Latin American countries and other low and middle-income countries (LMICs), which also have less well-developed lived experience advocacy movements.

While Brazil is not characterized by the frequent occurrence of natural disasters such as cyclones, earthquakes, tsunamis, or volcanoes, as in some other countries, rates of urban violence such as assaults, kidnappings, and murders are high [28]. Many victims also report fear, insecurity and lack of trust in authorities [5]. In many cases effective public policies to tackle such issues do not exist or are poorly implemented. This difference in context highlights the importance of cultural adaptation, which is reinforced by the finding that 25% of the items in the guidelines are newly suggested by the panels.

In Round 1, all items in following sections were endorsed by both panels: Actions to be taken immediately; When to seek professional help; and Children at large-scale traumatic events. This shows the panel’s agreement with preparing the first aider to act in potentially traumatic situations. It is known that such situations have an impact on all those involved, including professionals who also experience intense feelings of fear and helplessness [29]. This reinforces the importance of also focusing on the care of health professionals who are often first responders in traumatic situations [5, 6].

Both groups also endorsed strategies relating to the importance of first aiders working with and providing information to other professionals, perhaps reflecting a clear understanding that the role of the first aider is not to provide ongoing care but rather to provide the initial assistance, a concept that has been unfamiliar in some settings [30].

### Differences between Brazilian and English-language guidelines

Most items (110;83.9%) from the English-language guidelines were endorsed by both panels in the first round and 39 extra items suggested by the panel were endorsed in the second round, indicating the interest of panelists in the area and the need for cultural adaptation of such guidelines. Many of the suggestions of the Brazilian participants focused on the need to address the emotional and psychological needs of the affected person, rather than on the structural issues necessary for disaster response. The was reflected in items about the necessity to be welcoming, sympathetic and to listen carefully, without demanding or expecting answers. New items also focused on concern for the psychological aspects of the first aider, as well as the need to recognize their own limits and respect them.

This is different from some existing guidelines for responding to potentially traumatic events that rely more on collective health assistance, with interventions to physical aspects of disasters, to responses for families and communities, and assistance planning [17, 31]. This divergence can be understood by Brazil’s socioeconomic differences, with public policies and assistance services being more precarious, leading to a population with higher needs and less assistance [5, 12, 32]. In emergency situations—such as the accidents of Mariana and Brumadinho—the authorities were unable to provide sufficiently quick response, and years after the disaster the families still live with the consequences [12]. Thus, individuals must rely on each other rather than on community/government resources. These guidelines may therefore help to fill a gap in the design of interventions to help reduce such long-term psychological impacts.

### Differences between health professional and lived experience panels

While there were no strongly themed differences between panels, items relating to encouraging victims’ autonomy and strengthening their decisions were not recommended by professionals, although these items were somewhat more highly endorsed by people with lived experience. In contrast, in the Delphi study to develop English-language items about a person’s autonomy, decision about their professional help, and the need for the first aider to support their decisions were more strongly encouraged/endorsed. This has also been seen in other English language guidelines (e.g., the those for problem drinking [33]). This may be due to the paternalistic culture in Brazil, in which individuals usually expect some guidance from others instead of asserting their own independence [34]. Although this is driven by a legitimate intention to help, it may lead to a withdrawal of autonomy and lack of recognition of another person’s needs. This may create an understanding that those who experience trauma and disasters are passive victims, unable to have done anything to prevent the disaster. As such, the experience of trauma generates fear, impotence, and insecurity, making them further vulnerable and passive [15, 29]. Therefore, it is not expected that under these circumstances, people who have experienced potentially traumatic events will overcome and confront their difficulties with their own resources. Instead, it is expected that external interventions and policies are needed to help support and provide better conditions for them [5, 6].

### Strengths and limitations

One important strength of the study is the fact that it is the first cultural adaptation of mental health first aid for a person experiencing a potentially traumatic event for a Latin American country and may play a role in increasing the capacity of people to respond to such situations. This is of importance in Brazil, as a country with high urban violence, as well as other political and economic adversities, and a less well-resourced health system. In the current situation of the COVID-19 pandemic, it may also strengthen the capacity of individuals to support each other to intervene early and to encourage each other to seek professional help where available. The many suggestions made by Brazilian panelists who have had some contact with traumatic situations is another strength, ensuring inclusion of people with lived experience and an understanding of Brazilian culture. Moreover, the number of suggestions offered by participants was high, which provided sufficient content for generation of many additional items.

The relatively low retention rate of participants in round 2 is a limitation, particularly for the lived experience panel. This is likely to be because people with lived experience were more difficult to contact in the second round, possibly due to more limited access to the online questionnaire or to worsening mental health or life circumstances.

## Conclusion

This study involved the cultural adaptation for Brazil of the 2009 MHFA guidelines for helping someone who had experienced a potentially traumatic event. While there were many similarities with the English-language guidelines for high-income countries, the guidelines also incorporate actions of importance for Brazil, including the emphasis on the first aider’s management of the affected person’s psychological and emotional needs, rather than on structural or government responses to traumatic events.

The guidelines may be disseminated as a stand-alone product or used as the basis for MHFA training in Brazil, thus helping to improve knowledge and helping behaviours of the public towards people who have experienced potentially traumatic events. These guidelines may be particularly useful in the context of the recent COVID19 pandemic and the limited availability of mental healthcare resources in Brazil [35]. Future research should examine the impact of this.

## Electronic supplementary material

Below is the link to the electronic supplementary material.

Supplementary Material 1. Statements that were presented to the panels and their ratings across 3 rounds of the survey.

Supplement Material 2. Expert consensus guidelines for offering mental health first aid to a person after a potentially traumatic event in Portuguese.

Supplementary Material 3

## Data Availability

The data supporting our findings is attached as the Additional file, which contains all the statements that were presented to the panels and their endorsement rates.

## Abbreviations

MHFA: Mental Health First Aid
LMICs: Low- and middle-income countries
