# Supplementary material for: Cultural adaptation of the guidelines for offering mental health first aid to a person after a potentially traumatic event: a delphi expert consensus study in Brazil
Source: BMC Psychiatry. 2022 Oct 27;22:661. doi: 10.1186/s12888-022-04269-4 (PMC9609163; doi:10.1186/s12888-022-04269-4)
Supplement: Supplementary file 1 — Supplementary Material 1. Statements that were presented to the panels and their ratings across 3 rounds of the survey. [file 12888_2022_4269_MOESM1_ESM.pdf]

# Title page for supplementary file

## Cultural adaptation of the guidelines for offering mental health first aid to a person after a potentially traumatic event: a Delphi expert consensus study in Brazil

Kathlen Mendes[a], Carlos Henrique Mesquita Peres[a], Amanda Vidotto Cerqueira[a], Thais Alves Assumpção[a], Alexandre Andrade Loch[a,b], Nicola J Reavley[c]

*Corresponding author: Nicola J Reavley

[a] Laboratorio de Neurociencias (LIM 27), Instituto de Psiquiatria, Hospital das Clinicas HCFMUSP, Faculdade de Medicina, Universidade de Sao Paulo, Sao Paulo, SP, BR
[b] Instituto Nacional de Biomarcadores em Neuropsiquiatria (INBION), Conselho Nacional de Desenvolvimento Cientifico e Tecnológico, Brazil
[c] Centre for Mental Health, Melbourne School of Population and Global Health, University of Melbourne, Victoria 3010, Australia

Email addresses for all authors:
Kathlen Mendes: kathlen.mendes@gmail.com
Carlos Henrique Mesquita Peres: carlos.peres@fm.usp.br
Amanda Vidotto Cerqueira: amanda.vidotto@fm.usp.br
Thais Alves Assumpção: thais.assumpcao@fm.usp.br
Alexandre Loch: alexandre.loch@usp.br
Nicola Reavley: nreavley@unimelb.edu.au
