# Supplementary material for: Cultural adaptation of the guidelines for offering mental health first aid to a person after a potentially traumatic event: a delphi expert consensus study in Brazil
Source: BMC Psychiatry. 2022 Oct 27;22:661. doi: 10.1186/s12888-022-04269-4 (PMC9609163; doi:10.1186/s12888-022-04269-4)
Supplement: Supplementary file 2 — Supplement Material 2. Expert consensus guidelines for offering mental health first aid to a person after a potentially traumatic event in Portuguese. [file 12888_2022_4269_MOESM2_ESM.pdf]

Diretrizes de primeiros socorros de saúde mental para ajudar uma pessoa que passou por um evento potencialmente traumático

O que são situações traumáticas?

Trata-se de qualquer acontecimento que uma pessoa experencie ou testemunhe uma situação potencialmente violenta, que inclui:

Eventos causados pelo Homem	Eventos Naturais
Morte de uma pessoa	Deslizamento de terra
Ameaças a integridade física	Enchentes
Acidentes	Furacões
Violência física e/ou sexual	Tornados
Sequestros, assaltos	Queimadas

O termo potencialmente traumático refere-se aos impactos e danos que essas situações podem causar em uma pessoa. Nem todas reagem da mesma maneira, mas em casos graves, esses impactos podem ser geradores de ansiedade, depressão, transtorno de estresse agudo, transtorno pós-traumático, entre outras doenças mentais.

Para evitar esses danos emocionais e o desenvolvimento dessas e outras doenças, é fundamental que haja uma intervenção rápida e apropriada junto a essas pessoas. Ofertar acolhimento, cuidado e informação é imprescindível. As primeiras pessoas a ofertar isso, normalmente, são as pessoas da comunidade, amigos e familiares, ou seja, pessoas que estão próximas e chegam mais rápido para o socorro imediato. Porém, nem sempre elas possuem o conhecimento apropriado para isso.

Mas como oferecer ajuda? O que é preciso saber para ajudar nesse momento?

Este é um manual com orientações e sugestões de como abordar uma pessoa em situação de crise ou desastre. Ele foi desenvolvido para ajudar pessoas da comunidade a fornecer primeiros socorros a alguém que vivenciou algum evento traumático. O papel do socorrista é ajudar até que a ajuda profissional e especializada possa chegar e oferecer o serviço necessário, ou que a crise tenha se resolvido.

Que ações tomar imediatamente após o ocorrido?

Inicialmente veja se a pessoa está em segurança e se é possível conversar naquele momento. Se não há riscos que coloque a integridade física dela e a sua em perigo. E em caso afirmativo, retire imediatamente a pessoa e proporcione um local seguro.

É importante que se mantenha calmo dentro do possível e explique o que aconteceu e qual é o seu papel ali. Inclusive pergunte o nome da pessoa caso não conheça e passe a chamá-lo (a) pelo nome.

Assim que possível, averiguar quais são as necessidades básicas e providenciá-las o mais rápido possível. Observar os sinais de declínio do estado físico ou mental da pessoa e estar preparado para procurar assistência médica de emergência se necessário. Porém, não deve tentar assumir o papel dos profissionais especializados.

Caso a situação se trate de um crime, tenha ciência de que é preciso preservar o ambiente e possíveis provas, uma vez que poderá ocorrer coleta de evidências forenses (testes de swabs orais, evidências de roupas e pele).

Como se comunicar com a pessoa traumatizada?

Primeiramente mantenha uma linguagem clara, direta e objetiva, sem uso de termos técnicos ou difíceis de serem compreendidos e não tenha posição de superioridade. Esteja preparado para repetir sua fala o quanto for necessário caso a pessoa não entenda o que lhe é dito e esteja atento a comunicação não verbal.

Saiba que comportamentos de irritabilidade e mau humor podem ser uma resposta ao trauma sofrido, por isso não tome eles como uma forma de afronta pessoal. Seja amigável mesmo que a pessoa não se mostre receptiva a ajuda e demonstre que entende e se importa com a pessoa e a situação que ela passa. Sempre que possível seja sensível e acolhedor.

Entenda que a pessoa que sofreu um trauma, pode não se apresentar tão angustiada com o ocorrido quanto imagina, cada pessoa tem sua própria maneira de lidar com um trauma e você precisar estar receptivo a isso.

Encoraje a pessoa a falar sobre suas reações apenas se ela se sentir confortável com isso e lembre-se que dar apoio a alguém em situação pós trauma não precisa ser algo complicado e pode envolver pequenas coisas como fazer companhia para a pessoa, tomar uma xícara de chá ou café, conversar sobre coisas do dia a dia ou apenas dar um abraço.

Considere a sua saúde mental e a saúde mental da pessoa socorrida. Conhecer seus limites pessoais é essencial, inclusive saber a hora de parar de insistir.

Como conversar sobre o trauma?

Não force a pessoa a contar sua história, nem interrompa a fala da pessoa para compartilhar seus próprios sentimentos e opiniões, precisa saber reconhecer o seu momento de atuação e de fala. É possível que a pessoa precise falar repetidamente sobre o trauma e precisa estar disposto a ouvir.

Respeite o tempo da pessoa que se encontra fragilizada e acolha sua experiencia vivida e sua compreensão dela. Informe aos profissionais o maior número de informações para ajudar no seguimento do caso.

Evite dizer coisas que minimizem os sentimentos da pessoa, como "não chore", "fique calmo", ou coisas que minimizem a experiência vivida por ela, como "você deveria estar grato por estar vivo". Não faça promessas que não possa cumprir, como "vou te levar para casa em breve". Esteja ciente de que a pessoa pode estar vivenciando a "culpa do sobrevivente".

Qual assistência imediata para eventos traumáticos de larga escala?

Busque saber qual os serviços de emergências disponíveis no local e siga as instruções dadas pelos assistentes profissionais que trabalham no local. Forneça informações verdadeiras e, se for o caso, admita que carece das mesmas. Saiba as suas limitações de ajuda e acione a rede de apoio que melhor consiga atender as questões presentes.

Identifique as necessidades básicas como alimentação, abrigo, vestimentas e busque tentar atendê-las. Providencie conforto e dignidade da pessoa, como por exemplo, oferecer à pessoa algo para se cobrir (como um cobertor) ou pedir para que espectadores e mídia se afastem.

Respeite caso a pessoa não queira ter mais informações sobre o evento, mas informe a pessoa sobre quaisquer fontes de informações disponíveis que são oferecidas aos sobreviventes, por exemplo sessões informativas, fichas informativas ou números de telefone para linhas de informação.

Estratégias de enfretamento: Conversando

- Encoraje a pessoa a identificar fontes suporte e apoio, incluindo entes queridos e amigos.
- Encoraje a dizer aos outros quando elas necessitarem ou quiserem algo, ao invés de assumir que todos saberão o que ela precisa.
- Esteja atento aos riscos do afastamento de uma pessoa num período pós-traumático, a fim de evitar situações de grande risco identificando sinais como de planejamento ou ideação de suicídio e agressividade física, seja auto ou heteroagressão.
- Respeite o tempo e modo da pessoa.

- Identifique necessidades ao invés de cobrar posturas da vítima.

Estratégias de enfretamento: Ações

- Encoraje a pessoa a descansar quando estiver cansada.
- Encoraje a pessoa a passar um tempo em que se sinta segura e confortável.
- Auxilie a pessoa a encontrar fontes locais de apoio.
- Forneça informações à pessoa sobre os recursos disponíveis na comunidade (como por exemplo, telefones de emergência ou centros de saúde).

Lembre-se que a pessoa pode não se lembrar de todos os detalhes do evento ocorrido e ou pode repentina ou inesperadamente lembrar-se de detalhes do evento ocorrido.

Quando procurar ajuda profissional?

- Se a qualquer momento a pessoa apresentar comportamento suicida.
- Se os sintomas pós-trauma estiverem interferindo em suas atividades usuais por mais de 4 semanas.
- Se ela se sentir muito chateada ou temerosa por mais de 4 semanas.
- Se ela se sentir incapaz de escapar de sentimentos angustiantes intensos durante mais de 4 semanas.
- Se os resultados do trauma estiverem interferindo em suas relações pessoais (por exemplo, se a pessoa estiver se afastando de seus amigos e outras pessoas próximas) por mais de 4 semanas.
- Se ela estiver abusando de álcool ou outras drogas para lidar com o trauma.
- Sentir-se muito nervosa ou tiver pesadelos por causa ou sobre o trauma por mais de 4 semanas.
- Se ela não conseguir parar de pensar sobre o trauma por mais de 4 semanas.
- Se ela estiver impossibilitada de aproveitar a vida como resultado do trauma por mais de 4 semanas.

Se a pessoa não estiver à vontade com o profissional que a atendeu, você deve deixar claro para ela que pode tentar outro atendimento.

É necessário que haja um enfoque multidisciplinar no atendimento para este paciente, sobretudo no planejamento terapêutico psicológico e psiquiátrico. Assim, não cabe a você, que é o atendimento imediato, encarregar-se de ações em saúde posteriores ao evento.

Como ajudar a criança traumatizada?

Proteja a criança de outros possíveis danos e certifique-se que as necessidades físicas da criança como alimentação e lugar para dormir sejam atendidas E não julgue os sentimentos e pensamentos da criança.

Informe a criança que é normal se sentir chateada quando algo ruim ou assustador acontece. Não diga que alguém “foi dormir” quando esse alguém tiver falecido, pois isso pode causar medo de dormir na criança.

Não minta para a criança e nem faça promessas que não poderá cumprir. Perceba e respeite o tempo dela, exercitando um manejo sobre o que vai ser dito e como vai ser dito, sempre pensando na melhor forma de cuidado e acolhimento.

Garanta que você ou outro adulto estará disponível para cuidar da criança.

Como deve ser o atendimento com crianças em eventos traumáticos de larga escala?

Mantenha a criança junto com seus cuidadores ou entes queridos. Proteja a criança de imagens e áudios traumáticos, incluindo imagens da mídia. E não se comporte de forma que faça a criança vá se sentir em perigo.

Peça para que espectadores e a mídia fiquem longe da criança. Afaste a criança de pessoas muito aflitas (pessoas questão gritando, agitadas ou agressivas).

Tranquilize a criança, assegurando-lhe que ela não estará sozinha. Se necessitar deixar a criança sozinha por alguns minutos para atender outras pessoas, ele deve assegurar à criança que estará de volta o mais rápido possível.

Tente aparentar estar o mais calmo possível. E pergunte à criança o que faria ela se sentir melhor e mais segura. Não prometa nada nada que não se possa cumprir ou deixar claro que tentará algo que pode ou não acontecer.

Faça com que a criança se sinta segura, perto do possível, é essencial para o manejo da situação. Uma forma de acolher a criança é a abordagem de forma lúdica a fim de estabelecer um vínculo mais seguro.

Se comunicando com a criança traumatizada:

Converse com a criança utilizando linguagem e explicações apropriadas à idade dela. Esteja ciente que a criança pode parar de falar completamente depois de um trauma e se isso ocorrer, não deve tentar forçar ou coagir a criança a falar.

Não minta e nem engane a criança em nenhum momento. Não faça nada que possa coagir a criança a falar sobre seus sentimentos ou memórias do trauma antes que elas se sintam confortáveis para fazê-lo. E nem falar sobre o trauma antes de estar pronta.

Permita que a criança pergunte e deve responder às questões da forma mais verdadeira possível. Se a criança souber detalhes verdadeiros e perturbadores, não negue eles.

Seja paciente se a criança questionar a mesma coisa diversas vezes. E permita que a criança fale, escreva ou desenhe sobre seus sentimentos. Deixe que expresse suas emoções através de brincadeiras. Inclusive, encoraje criança a fazer coisas que ela gosta (como brincar com brinquedos, ler livros).

Não diga à criança como ela deveria ou não deveria estar se sentindo e nem fale para a criança ser forte ou para não chorar.

Não se irrite se a criança demonstrar emoções intensas, ao contrário demonstre que você entende e se importa. Diga que fará o melhor para mantê-la segura.

E se o socorrista mora com a criança traumatizada?

- Seja o mais previsível possível e não fique irritado, crítico ou chame a criança de "bebê" se esta começar a urinar na cama, se comportar mal ou chupar o dedo.
- Não atue a longo prazo como um apoio psicológico, pois este deve ser realizado por uma equipe que tenha tempo hábil a dedicar para as vítimas de traumas.
- Importante a criança ter um adulto de referência a quem possa recorrer com suas angústias e medos após o trauma.
- Sobre a criança se sentir no controle, é importante, mas também se faz necessário mostrar que ela pode se permitir ser cuidada, pode se permitir ser criança.

Como lidar com comportamentos de evitação e birras?

Saiba que a criança pode evitar coisas que lembrem o trauma (como lugares específicos, estar em carros, pessoas específicas ou a separação de seus cuidadores). Por isso tente descobrir quais são os gatilhos para o medo repentino e regressão em relação ao trauma na criança. E se ela evitar coisas que a lembram do trauma, mas não parecer muito angustiada, você deve reasseguRá-la de que ela está segura.

Observe a reação da criança quando fala do trauma e de liberdade para a criança se expressar a qualquer momento. Se possível encontrar um suporte psicológico para a criança.

Entenda a importância da comunicação não violenta neste processo.

Quais as questões legais relacionadas ao abuso infantil?

Conheça as leis ou regulações locais sobre denúncias de suspeita de abuso infantil. Ofereça segurança, confiança e encaminhe sempre para serviços especializados. Também esteja atento e compreenda a verdade da criança, sem menosprezar ou duvidar. Mantenha a criança em um lugar seguro e longe do agressor.

Se a criança relatar algum tipo de abuso, contate as autoridades competentes e procure aconselhamento especializado imediatamente. Procure trabalhar em conjunto com as autoridades competentes para garantir a segurança dela.

Se a criança relatar abuso, mantenha a calma e tranquilize a criança ao dizer que ela fez a coisa certa ao falar. Proporcione a criança o maior conforto possível para falar sobre ocorrido. Deixe claro para a criança de que o abuso não foi culpa dela e que você acredita no que ela relatou.

Como obter ajuda profissional para a criança traumatizada?

- Se a qualquer momento a criança apresentar comportamento suicida, o(a) socorrista deve buscar ajuda profissional imediatamente.
- Se ela demonstrar reações súbitas graves ou tardias ao trauma por duas semanas ou mais.
- Se os sintomas pós-trauma estiverem interferindo nas atividades usuais dela por mais de duas semanas.
- Se estiver incapaz de lidar com sentimentos angustiantes por mais de duas semanas.
- Se as relações pessoais dela estiverem abaladas (por exemplo, se ela se afasta de seus cuidadores ou amigos) em decorrência do trauma por mais de duas semanas.
- Se estiver incapacitada de aproveitar a vida em decorrência do trauma por mais de duas semanas.
- Se estiver se sentindo muito chateada ou com muito medo por 4 semanas ou mais.
- Se estiver agindo de forma muito diferente por mais de 4 semanas depois do trauma.
- Se sentir muito nervosa ou tiver pesadelos por causa do trauma ou sobre o trauma por mais de 4 semanas.
- Se não conseguir parar de pensar sobre o trauma por mais de 4 semanas.
- Se ela fizer birras, apresentar muito medo ou apresentar choros intensos em resposta evitativa a algo que a lembra do trauma por mais de 4 semanas.

Esteja ciente dos tipos de ajuda profissional disponíveis para a criança.

Esteja ciente dos sintomas associados com o trauma podem aparecer inesperadamente meses ou anos depois do evento e, que se isso ocorrer, pode ser necessário procurar ajuda profissional.

Quando for contatar alguma autoridade sobre abuso, relatar tudo o que a criança descrever.

Atente-se para o comportamento dos responsáveis em cuidar da criança.

Não precisa esperar o tempo de semanas para encaminhar para ajuda profissional, a criança deve ser encaminhada a ajuda profissional (psicológica) imediatamente.

É importante que o socorrista também tenha auxílio psicológico para lidar com seus medos, inseguranças e possíveis consequências daquele auxílio ao menor.

Desenvolvimento Guideline

Esse é um manual de primeiros socorros, com recomendações de cuidados básicos para intervir diante de situações de crise e/ou traumáticas. As diretrizes foram baseadas nas diretrizes em língua inglesa, que foram fundamentadas no conhecimento de pessoas que experenciaram situações de crises/traumas (vítimas e cuidadores) e profissionais de saúde mental (médicos, pesquisadores, psicólogos, profissionais de saúde) da Austrália, Canadá, Alemanha, Irlanda, Países Baixos, Nova Zelandia, Suécia, Suíça, Reino Unido, Estados Unidos da América. Estas foram então adaptadas culturalmente com base nas opiniões dos profissionais de saúde brasileiros e de pessoas com experiência vivida. Detalhes sobre a metodologia podem ser encontrados aqui: Mendes KN, Mesquita Peres CH, Vidotto Cerqueira A, Alves Assumpção T, Andrade Loch A, Reavley NJ: Adaptação cultural das diretrizes para oferecer primeiros socorros à saúde mental após um evento potencialmente traumático: um estudo de consenso de peritos Delphi no Brasil (em submissão). 2021

Como usar esse Guideline

É importante que você adeque essas orientações as necessidades das pessoas que estão recebendo ajuda e do momento vivido, pois se trata apenas de um conjunto de recomendações gerais.

Embora esses guidelines tenham direitos autorais, eles podem ser livremente reproduzidos para fins não comerciais, desde que a fonte seja citada. Por favor, cite esse guideline da seguinte forma: Diretrizes para oferecer primeiros socorros em saúde mental a uma pessoa após um evento potencialmente traumático no Brasil. Mental Health First Aid Programa de Treinamento e Pesquisa, Universidade de São Paulo e Universidade de Melbourne 2022.

[Referência]

Questionamentos devem ser enviados para: alexandre.loch@usp.br / nreavley@unimelb.edu.au
